# Therapeutic potential of a novel virulent bacteriophage XQ-1 against avian pathogenic *Escherichia coli* infection in broiler chickens

**DOI:** 10.1016/j.psj.2026.107600

**Published:** 2026-08-12

**Authors:** Yuqing Duan, Changchun Gao, Xi Yang, Pei Zhu, Jinping Wang, Yun Zhou, Zihan Yang, Yitong Guo, Guangming Tian, Jiaying Liu, Xiaowei Fang, Lei Tan, Mengting Zuo, Jun Yao

**Affiliations:** a College of Animal Science and Technology, Yangtze University, Jingzhou 434025, Hubei, China; b Beijing Huadu Yukou Poultry Industry Co., Ltd., Beijing, 102206, Beijing, China; c Yunnan Tropical and Subtropical Animal Virus Diseases Laboratory, Yunnan Animal Science and Veterinary Institute, Kunming, 650224, Yunnan, China; d Jingzhou District Animal Husbandry Development Center, Jingzhou, 434025, Hubei, China; e Hunan Provincial Key Laboratory of the TCM Agricultural Biogenomics, Changsha Medical University, Changsha, 410208, Hunan, China

**Keywords:** APEC, Bacteriophage, Biological characteristics, Genome characteristics, Bacteriophage therapy

## Abstract

Avian Pathogenic *Escherichia coli* (APEC) represents a significant subgroup within extraintestinal pathogenic *Escherichia coli* strains and constitutes a substantial threat to the global poultry industry. Although the negative impacts of APEC have been mitigated considerably through antibiotic use, this practice has concurrently facilitated the widespread emergence and dissemination of antibiotic-resistant APEC strains worldwide. Consequently, bacteriophage has emerged as a promising alternative to antibiotics. In the current study, a novel virulent bacteriophage, designated XQ-1, was isolated from a sewage sample collected at a broiler chickens farm in Hubei Province, China. Notably, this bacteriophage exhibited the capability to lyse multiple APEC strains, including O1, O2, and O78 serotypes. The optimal multiplicity of infection (MOI) for bacteriophage XQ-1 was determined to be 0.001, yielding a maximum viral titer of 4.73 ± 0.31 × 10^11^ plaque-forming units (PFU) per milliliter. This bacteriophage displayed a latent period of 30 min, and a burst period of 80 minutes, corresponding to a burst size of 348 PFU per infected cell. Additionally, bacteriophage XQ-1 retained high lytic activity across a temperature range of 4–50 °C and maintained tolerance with a pH range of 3–11. Further in vitro studies demonstrated that bacteriophage XQ-1 holds potential as an effective disinfectant capable of directly lysing APEC strain JZ-1. In chick models subjected to intraperitoneal injection, exposure to APEC resulted in 100% mortality in chicks. However, treatment with bacteriophage XQ-1 significantly improved the survival rates of infected broiler chickens, reduced organ indexes, decreased bacterial loads in the liver and heart organs, and mitigated intestinal damage caused by APEC infection. Collectively, these findings suggest that bacteriophage XQ-1 represents a promising candidate for the prevention and treatment of APEC infections in poultry.

## Introduction

Avian Pathogenic *Escherichia coli* (APEC) is a subgroup within extraintestinal pathogenic *Escherichia coli* strains that can infect various avian species, leading to either localized or widespread infections (Soleymani et al., 2020). The clinical symptoms of APEC infection in chickens primarily include reproductive tract infections, omphalitis, hepatitis, pericarditis, peritonitis, and arthritis (Wang et al., 2024; Xu et al., 2026). Consequently, the widespread occurrence of this disease causes significant economic losses to the poultry industry globally. APEC is widespread in China, with more than twenty-five serotypes identified (Nawaz et al., 2024). Among these, the three most common serotypes are O1, O2, and O78, and are associated with high fatality rates in chickens (Yao et al., 2023; Li et al., 2025). The prevention and management of APEC infections have traditionally relied on the use of antibiotics. However, the inappropriate use of antibiotics subsequently leads to a higher occurrence of multidrug-resistant APEC strains (Wintachai et al., 2024). In light of this, China has fully prohibited the use of growth-promoting antibiotics in animal feed, imposed strict limits on therapeutic drugs, encouraged the use of alternative products, and enhanced comprehensive supervision throughout the entire supply chain (Wen et al., 2022; Zheng et al., 2025).

Bacteriophages, also called phages, are commonly found in the environment, exhibit specificity for their hosts, and can selectively infect and destroy pathogenic bacteria without affecting other beneficial microbial communities (Islam et al., 2020; Yeager et al., 2024). Bacteriophages were first discovered in 1915, and later were used to treat bacterial infections in both poultry and humans (Abedon et al., 2011). However, research focused on bacteriophages has greatly declined because of the widespread use of antibiotics in medical treatment (Rahman et al., 2026). In recent years, there has been a growing interest in using bacteriophages to control bacterial infections in various animal species (Desiree et al., 2021; Metsemakers et al., 2023; Rahman et al., 2026). In particular, several studies have identified bacteriophages that specifically target APEC strains (Yao et al., 2023; Jhandai et al., 2024; Huang et al., 2025; Li et al., 2025); however, their biological characteristics, especially their potential use as disinfectants and therapeutic agents, have not been thoroughly investigated.

In this study, a virulent bacteriophage named XQ-1 was successfully isolated from wastewater at a chicken farm in Jingzhou city, Hubei Province, China. Its fundamental biological properties and genetic characteristics were then investigated. Notably, its potential use as a novel microbial disinfectant and therapeutic agent against APEC infection in chicken models was assessed.

## Material and methods

### Sample collection and APEC isolation

A total of 103 fecal samples and wastewater samples were collected from 47 chicken farms (2∼3 samples from each farm) located in Hubei, Hunan, and Guizhou Provinces between 2024 and 2025. Briefly, fresh fecal specimens (3∼5 g each, *n* = 73) were obtained utilizing sterile swabs, while wastewater samples (30∼40 mL each, *n* = 30) were gathered in sterile centrifuge tubes. All samples were conveyed to the laboratory in ice boxes within a six-hour timeframe. Upon receipt, fecal samples (1 g) were homogenized in 9 mL of sterile phosphate-buffered saline (PBS); wastewater samples were subjected to centrifugation, and the resulting pellets were subsequently resuspended in PBS. The supernatant of each sample was initially cultured in LB broth (Sangon Biotech, Shanghai, China) and then plated onto MacConkey agar (Sangon Biotech, Shanghai, China). The pink colonies (at least 3 colonies for each sample) on the MacConkey agar were further confirmed as *E. coli* through molecular identification using PCR targeting a specific *E. coli* gene. Using a similar approach, another PCR test was conducted to verify the presence of APEC by using APEC-specific primers. In addition, the serotypes of the isolated APEC strains were confirmed by PCR with primers targeting O1, O2, O78, and O145 serogroups (Li et al., 2010).

### Isolation and purification of bacteriophage XQ-1

Among the above collected samples, Fecal (*n* = 12) and wastewater (*n* = 8) samples collected from seven chicken farms in Jingzhou city were used for bacteriophage isolation. First, the fecal and wastewater samples from one farm were mixed with PBS and then centrifuged at 5000 rpm for 10 min. The supernatant was filtered through a 0.22 μm membrane filter and subsequently mixed with 10 mL of Lysogeny Broth broth containing 10^6^ Colony-Forming Unit of the APEC strain isolated from the respective farm, 1 mM MgCl_2_, and 1 mM CaCl_2_. This mixture was incubated overnight at 37°C in a incubator. The supernatant was filtered again through a 0.22 μm filter to obtain the crude phage lysate.

In the next step, bacteriophages were isolated and purified following the techniques described by Xu et al. (2026). In brief, the bacteriophage solution was diluted to a series of tenfold dilutions ranging from 10 ^to 1^ to 10^-10^. Each diluted bacteriophage solution was mixed with 10^8^ CFU of corresponding targeted APEC bacterial suspension, then combined with 5 mL of semi-solid medium to prepare semi-solid plates. The plates were incubated overnight at 37 °C. The next day, individual plaques were picked, and a single was selected and isolated for plaque purification using the same method (Xu et al., 2026). To ensure the acquisition of a genetically homogeneous phage isolate, this purification process was repeated at least three times.

### Morphological observation of bacteriophage XQ-1

Considering the biological properties of the isolated bacteriophages, one novel bacteriophage, designated as XQ-1 isolate, was chosen for further experimental investigations. A 50 mL portion of the purified phage stock was ultracentrifuged at 50,000 rpm and 4 °C for 30 min. The supernatant was discarded, and the precipitate was resuspended in 100 μL of PBS by vortexing. Next, 20 μL of this suspension was placed onto a copper grid and allowed to adsorb for 20 min. The excess liquid was blotted away with filter paper. Finally, 5 μL of the staining solution was applied to grid with 2% phosphotungstic acid for 2 min, air dried, and examined using an electron microscope.

### Whole genome sequencing and genetic analysis

The genome of bacteriophage XQ-1 was extracted according to the protocol provided with the viral genome DNA/RNA extraction kit (Tiangen Biochemical Technology, Beijing, China). The quality and purity of the extracted genome were assessed using agarose gel electrophoresis and concentration measurements. Genome samples which met the quality criteria were sent for sequencing using the Illumina NovaSeq 6000 platform at Shanghai Biozeron Biotechnology Co, Ltd. (Shanghai, China).

The genome of phage XQ-1 was assembled with ABySS (version 2.2.0) and CapCloser (version 1.12) (Simpson et al., 2009; Xu et al., 2020). Potential open reading frames (ORFs) within the genome were predicted using ORF finder. These predicted ORFs were then selected for functional annotation analysis using the BLASTP algorithm. To evaluate its potential safety risks, the genome was analyzed for virulence and antibiotic resistance genes using the Virulence Factor Database and the Comprehensive Antibiotic Resistance Database, respectively. Moreover, the genomic map of phage XQ-1 was created using Proksee (http://proksee.ca/). The complete genomes of APEC bacteriophage strains were downloaded from the NCBI database, and a phylogenetic tree was constructed using on their complete genomes with the neighbor-joining method (Kimura 2-parameter model, 1000 bootstrap replicates) in MEGA 8.0 software.

### Assessment of bacteriophage host range

A Double-layer assay was performed to assess the host range of the bacteriophage XQ-1 isolates (Bilhman et al., 2025). This experiment included a total of 67 APEC strains, two Salmonella ioslates from broiler chickens, one chicken-source Staphylococcus aureus, and two chicken-source Bacillus strains. In brief, each of the isolated bacteria was separately incubated into LB liquid medium for amplification. Then, 100 μL of each bacterial culture was spread onto LB agar plates. After the bacterial suspension dried, 10 μL of purified bacteriophage XQ-1 was applied onto the plate. The plates were inverted and incubated overnight at 37 °C. The lytic activity of bacteriophage XQ-1 on each bacterial strain was determined by observing the presence or absence of plaques on the plates.

### Evaluation of the stability of bacteriophage XQ-1 under different temperature and pH conditions

To evaluate the temperature tolerance of bacteriophage XQ-1, the phage XQ-1 was diluted to a final concentration of 10^9^ PFU/mL. Then, 1 mL of this bacteriophage solution (containing 10^9^ PFU) was incubated in water baths set at 37 °C, 50 °C, 60 °C, and 70 °C for one hour (Toaquiza-Vilca et al., 2025). After incubation, the viral titer was measured using the double-layer agar approach.

To assess its pH stability, the pH value of LB medium was adjusted between 3 and 14 by utilizing HCl or NaOH. 10 μL of purified bacteriophage XQ-1 (10^9^ PFU) was added into 990 μL of LB medium with different pH value, and incubated at 37°C for one hour (Xu et al., 2026). The phage titer in each group was analyzed using the double-layer agar method.

### Determination of the one-step growth curve of bacteriophage XQ-1

A 200 μL phage solution containing 1 × 10^9^ PFU was combined with 200 μL of host bacteria containing 1 × 10^10^ CFU and incubated with shaking at 37 °C for 10 min. The mixture was then centrifuged at 5000 rpm for 10 minutes, the supernatant was removed, and the pellet was resuspended in 5 mL of liquid LB medium. A 200 μL portion of this concentrated suspension was centrifuged at 12,000 rpm for 30 s, and the phage titer in the supernatant was measured, marking this as time zero at 37 °C. The remaining culture was incubated with shaking at 37 °C, and samples were taken at 5, 10, 15, and 20 min, followed by additional samples every 10 minutes up to 120 minutes (at 30, 40, 50, 60, 70, 80, 90, 100, 110, and 120 min). Phage titers were determined using the double-layer agar technique at each time point (Xu et al., 2026). A one-step growth curve was then created by plotting the phage titer (PFU/mL) against time to analyze the phage replication cycle.

### Determination of the adsorption activity of bacteriophage XQ-1

A reaction mixture with a total volume of 1 mL was prepared in a 1.5 mL microcentrifuge tube. This mixture comprised 100 μL of host bacterial culture at a concentration of 10¹⁰ CFU/mL (resulting in a final concentration of 10⁹ CFU/mL), 100 μL of phage stock at 10⁹ PFU/mL (yielding a final concentration of 10⁸ PFU/mL), and 800 μL of liquid LB medium. The mixture was incubated at 37 °C using either a water bath or an incubator. Aliquots of 100 μL were collected at 0, 2, 4, 6, 8, 10, and 12 min following the initiation of incubation. The 0-min sample was filtered within 15 s of mixing to serve as the baseline measurement of total phage input. All samples collected were immediately subjected to centrifugation for subsequent determination of phage titer (Xu et al., 2026).

### In vitro disinfectant evaluations of bacteriophage XQ-1 targeting the APEC JZ-1 strain

#### Efficacy of decontaminating APEC JZ-1 from artificially contaminated utensils

A 12-well plate was inoculated with 100 μL of the APEC JZ-1 strain with a final concentration of 10^6^ CFU per well. After the liquid had dried, the wells were treated with 100 μL of the bacteriophage XQ-1 strain at a final concentration of 10^4^ CFU per well, ensuring that the entire area containing the bacteria was completely covered by the bacteriophage solution. Meanwhile, bacteria samples incubated with PBS without bacteriophage XQ-1 or antibiotics (Penicillin, 100 U/mL and Streptomycin, 100 μg/mL) were used as negative and positive controls, respectively. Following a 10 min incubation at either 37 °C or 4 °C, 500 μL of LB broth was added to each well. On one hand, 10 μL of the mixture was serially diluted and subsequently plated onto LB agar medium, followed by incubation at 37 °C in a temperature-controlled incubator overnight. On the other hand, 10 μL of the mixture was inoculated into 3 mL of LB liquid medium and cultured with shaking at 200 rpm in a 37 °C incubator for six hours. Bacterial growth was then assessed by measuring the optical density at 600 nm (OD_600_) for each sample group.

#### Efficacy of decontaminating APEC JZ-1 from artificially contaminated eggshells

A total of 48 specific pathogen-free chicken eggs (Jinghong No.1) were obtained from Yukou Poultry Industry Co., Ltd., Hubei, and then were divided into six groups, with eight eggs in each group. To simulate external environmental contamination, the eggshell surface on each egg was artificially contaminated by depositing 50 μL of the APEC JZ-1 suspension (final concentration of 10^7^ CFU per egg) onto the intact shell surface (without puncturing the shell). After allowing the liquid to dry, bacteriophage suspension contained 10^9^ PFU/mL was sprayed onto the contaminated eggshell area until runoff using a spray bottle. Meanwhile, a PBS solution without bacteriophage was set as controls. After incubation periods of 0, 12, and 24 h, the eggs were rinsed with 2 mL of PBS, and 100 μL of the washing solution was collected to determine the bacterial concentration.

### In vivo experiments evaluating the antibacterial activity of phage XQ-1 against APEC JZ-1 strain in chicken model

The animal experiments were performed in compliance with the Chinese guidelines for Animal Welfare and received approval from the Animal Ethics Committee of Yangtze University (Approval No. 202601011). A total of 72 one-day-old white-feathered, clean-grade broiler chickens were individually housed in cages measuring 300 mm × 200 mm × 200 mm. After one week, these cages were randomly assigned to six groups (*n* = 12 per group) and treated as follows (Table 1): Group A (blank control) received sterile physiological saline (200 μL/broiler chickens, i.p.); Group B (APEC control) received APEC JZ-1 strain (1 × 10⁹ CFU/broiler chickens, i.p.); Group C (phage control) received phage XQ-1 (1 × 10^9^ PFU/broiler chickens, i.p.); Group D (high-dose phage treatment) received equal volume of sterile physiological saline containing phage XQ-1 (1 × 10^9^ PFU/broiler chickens, i.p.) and APEC JZ-1 (1 × 10^7^ CFU/broiler chickens, i.p.). Group E (low-dose phage treatment) received phage XQ-1 (1 × 10^7^ PFU/broiler chickens, i.p.) and APEC JZ-1 (1 × 10^7^ CFU/broiler chickens, i.p.), simultaneously. Group F (antibiotic treatment) received APEC JZ-1 (1 × 10⁷ CFU/broiler chickens, i.p.) and oral florfenicol (20 mg/kg). All cages were kept under identical conditions with a 12 h light/dark cycle, and the broiler chickens had unlimited access to food and water during the experiment.

**Table 1:** Experimental grouping and dosage protocol for animal studies.

Group (*n* = 12)	Treatment (intraperitoneal injection)
Group A (NaCl, Blank control)	Physiological saline (200 μL per animal) was administered.
Group B (XQ-1)	Equal volume of physiological saline containing bacteriophage XQ-1 (1 × 10^9^ CFU per animal) was administered.
Group C (JZ-1)	Equal volume of physiological saline containing APEC JZ-1 (1 × 10^9^ PFU per animal) was administered.
Group D (JZ-1+XQ-1, high)	Equal volume of physiological saline containing bacteriophage XQ-1 (1 × 10^9^ PFU per animal) and APEC JZ-1 (1 × 10^7^ CFU per animal) was administered.
Group E (JZ-1+XQ-1, low)	Equal volume of physiological saline containing bacteriophage XQ-1 (1 × 10^7^ PFU per animal) and APEC JZ-1 (1 × 10^7^ CFU per animal) was administered.
Group F (E+A)	Chickens were simultaneously administered Florfenicol orally at a dosage of 20 mg/kg and intraperitoneally inoculated with the APEC JZ-1 (1 × 10^9^ CFU per animal).

On the seventh day of the challenge, the survival rates of broiler chickens in each group were assessed, after which all broiler chickens were humanely euthanized by cervical dislocation following deep anesthesia with sodium pentobarbital (40 mg/kg. i.p.). Samples of feces, heart, liver, and intestine were collected from each group for further analyses: (1) the heart and liver samples were measured to calculate their respective organ indices; (2) Approximately one gram of heart, liver, or fecal tissue was weighed, and homogenized in 10 mL of cold PBS, respectively. The resulting homogenates were subjected to a tenfold dilution and subsequently plated onto MacConkey agar. The agar plates were incubated overnight at 37°C, after which bacterial colonies were enumerated to quantify the titer of APEC. Selected colonies were picked and analyzed using a targeted PCR assay to confirm the presence of APEC. (3) the ileum tissues were fixed in 4% paraformaldehyde and submitted to Wuhan Servicebio Technology Co., Ltd (Wuhan, China) for histopathological analysis.

### Statistical Analysis

All experiments were independently performed with more than three biological replicates, and the data are shown as the mean ± standard deviation (SD) form. Statistical analysis was performed utilizing Student’s t-test in GraphPad Prism 8.0. The significance levels between different groups were considered as follows: *P < 0.05; **P < 0.01; ***P < 0.001.

## Results

### Isolation of APEC and serotype determination by PCR

**Fig. 1:**
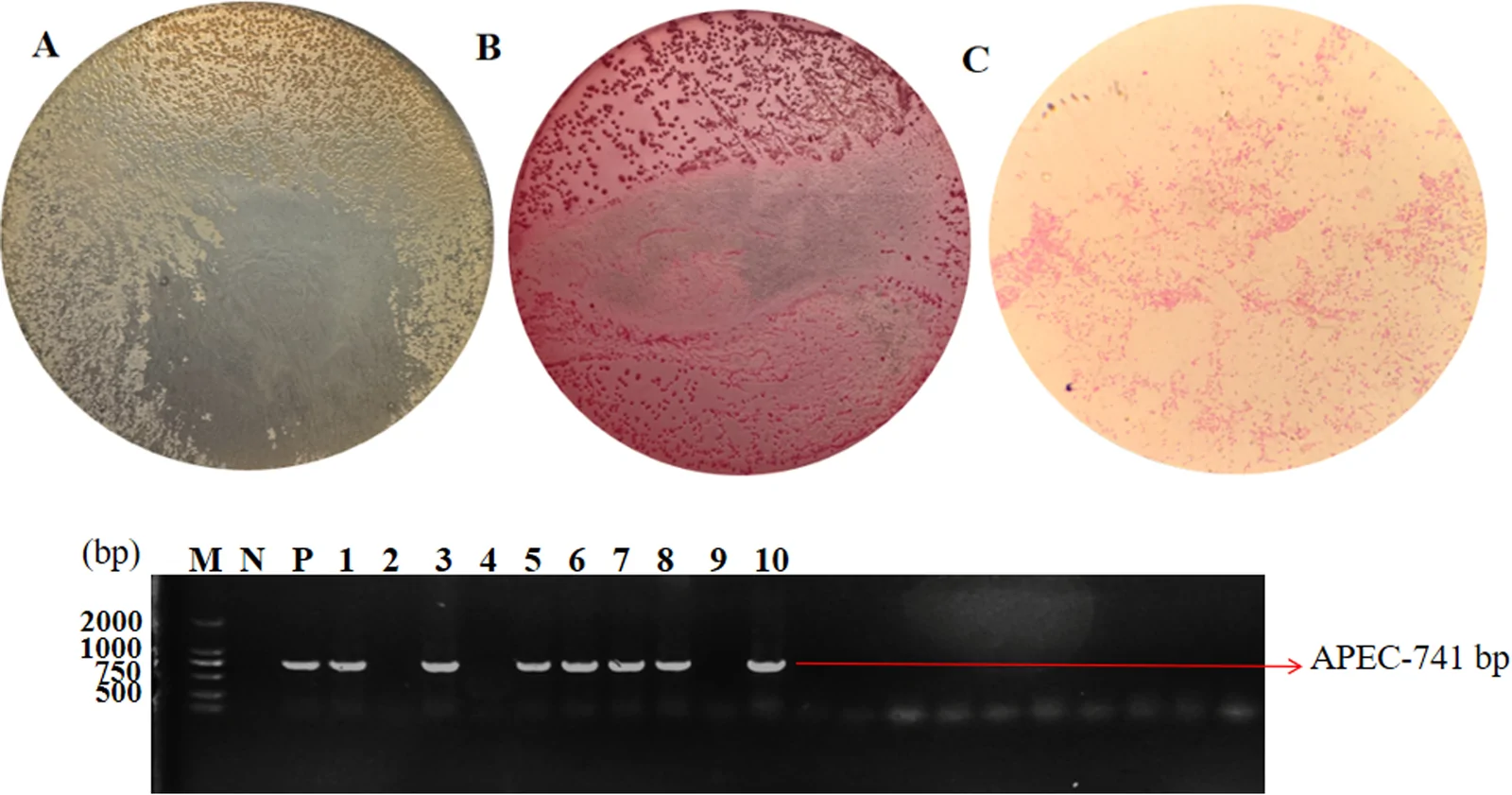
Isolation and molecular identification of APEC by PCR. Isolation of bacteria on LB and MacConkey agar media (A-B), Gram staining analysis (C), and identification of APEC using specific PCR (D).

A total of 103 fecal and liver tissue samples were collected from both clinically sick and healthy broiler chickens in Hubei Province for detecting APEC. Following initial bacterial isolation (Fig. 1A and B), Gram staining (Fig. 1C), and a targeted PCR assay specific for *E. coli* were performed (Fig. 1D). The findings showed *E. coli* was detected in 74.8% (77/103) of the samples collected from 41 chicken farms. Subsequent PCR assays specific for APEC identified 67 strains (65.00%, 67/103) as APEC, which were originated from 39 farms. Further serotype identification via PCR analysis showed that 13 APEC strains were classified as serotype O1, 9 strains as serotype O2, and 11 strains as serotype O78, where the remaining strains (*n* = 34) were not associated with any of these three serotypes (Table 2).

**Table 2:** Evaluation of the bacteriolytic effectiveness of five distinct bacteriophage strains.

Number	Strain	Isolation region	Source	Species	Serotype	XQ-1	XQ-2	XQ-3	XQ-4	XQ-5
1	JZ-1	Jingzhou	Chicken	APEC	O2	+	-	+	-	-
2	J-21	Jingzhou	Chicken	APEC		+	-	-	-	+
3	J-24	Jingzhou	Chicken	APEC	O78	+	-	+	-	-
4	J-31	Jingzhou	Chicken	APEC		-	-	-	-	-
5	J-41	Jingzhou	Chicken	APEC	O2	+	-	-	+	-
6	J-42	Jingzhou	Chicken	APEC	O2	-	+	-	-	-
7	Z1-21	Yichang	Chicken	APEC	O78	-	-	-	-	-
8	Z1-22	Yichang	Chicken	APEC		+	-	-	-	-
9	Z1-32	Yichang	Chicken	APEC	O2	+	-	-	-	-
10	Z1-41	Yichang	Chicken	APEC		-	-	-	-	-
11	Z2-12	Yichang	Chicken	APEC	O78	+	-	-	-	-
12	Z2-31	Yichang	Chicken	APEC		-	-	-	-	-
13	Z2-42	Yichang	Chicken	APEC	O2	+	-	+	-	-
14	Z3-11	Yichang	Chicken	APEC		-	-	-	-	-
15	Z3-12	Yichang	Chicken	APEC	O78	-	-	-	-	-
16	Z3-14	Yichang	Chicken	APEC		-	-	-	-	-
17	Z3-32	Yichang	Chicken	APEC	O1	+	-	+	-	-
18	Z3-42	Yichang	Chicken	APEC	O1	+	-	-	-	-
19	Z4-11	Yichang	Chicken	APEC	O1	-	-	-	-	-
20	Z4-21	Yichang	Chicken	APEC	O1	+	-	-	-	-
21	Z4-22	Yichang	Chicken	APEC	O1	-	-	-	-	-
22	Z4-31	Yichang	Chicken	APEC	O1	-	-	+	-	-
23	Z4-42	Yichang	Chicken	APEC	O1	+	-	-	-	-
24	Z5-11	Yichang	Chicken	APEC	O78	+	-	-	-	-
25	Z5-21	Yichang	Chicken	APEC	O78	+	-	-	-	-
26	Z5-22	Yichang	Chicken	APEC	O1	+	-	-	-	-
27	Z5-32	Yichang	Chicken	APEC	O1	+	-	-	-	-
28	Z5-41	Yichang	Chicken	APEC		+	-	-	-	-
29	L1-2	Hunan	Chicken	APEC		+	-	+	-	-
30	L2-2	Hunan	Chicken	APEC	O78	+	-	-	-	-
31	W1-1	Hunan	Chicken	APEC		-	-	-	-	-
32	W1-2	Hunan	Chicken	APEC	O78	-	-	-	-	-
33	W2-1	Hunan	Chicken	APEC	O78	-	-	-	-	-
34	W2-2	Hunan	Chicken	APEC		-	-	-	-	-
35	X1-1	Hunan	Chicken	APEC		+	-	+	-	-
36	X2-1	Hunan	Chicken	APEC		+	-	-	-	-
37	X2-2	Hunan	Chicken	APEC		-	-	-	-	-
38	Y1-1	Hunan	Chicken	APEC	O78	+	-	+	-	-
39	Y1-2	Hunan	Chicken	APEC		+	-	-	-	-
40	Y2-2	Hunan	Chicken	APEC		+	-	-	-	-
41	Y1	Jingzhou	Chicken	APEC	O78	-	-	-	-	-
42	JY-1	Jingzhou	Chicken	APEC		-	-	-	-	-
43	1-2-1	Jingzhou	Chicken	APEC	O1	+	-	-	-	-
44	1-2-2	Jingzhou	Chicken	APEC	O1	+	-	-	-	-
45	1.18	Guizhou	Chicken	APEC	O1	-	-	-	-	-
46	2.13	Guizhou	Chicken	APEC		-	-	-	-	-
47	2.17	Guizhou	Chicken	APEC		-	-	-	-	-
48	2.20	Guizhou	Chicken	APEC	O1	-	-	-	-	-
49	2.22	Guizhou	Chicken	APEC		-	-	-	-	-
50	2.28	Guizhou	Chicken	APEC		-	-	-	-	-
51	3.8	Guizhou	Chicken	APEC	O2	-	-	-	-	-
52	3.26	Guizhou	Chicken	APEC		-	-	-	-	-
53	3.27	Guizhou	Chicken	APEC		+	-	-	-	-
54	Q-11	Jingzhou	Chicken	APEC		+	-	-	-	-
55	Q-31	Jingzhou	Chicken	APEC	O2	-	-	-	-	-
56	Q-32	Jingzhou	Chicken	APEC		+	-	-	-	-
57	Q-41	Jingzhou	Chicken	APEC		+	-	-	-	-
58	JX-A	Jingzhou	Chicken	APEC		-	-	-	-	-
59	JX-B	Jingzhou	Chicken	APEC		-	-	-	-	-
60	Z-1	Xiangyang	Chicken	APEC		-	-	-	-	-
61	SX	Xiangyang	Chicken	APEC	O2	-	-	-	-	-
62	XJ-2	Xiangyang	Chicken	APEC		+	-	-	-	-
63	XJ-5	Xiangyang	Chicken	APEC		-	-	-	-	-
64	GF2-1	Guizhou	Chicken	APEC	O2	-	-	-	-	-
65	GF3-1	Guizhou	Chicken	APEC		+	-	-	-	-
66	CS-1	Guizhou	Chicken	APEC		+	-	-	-	-
67	CS-2	Guizhou	Chicken	APEC		+	-	-	-	-
68	SM-1	Jingzhou	Chicken	Salmonella		-	-	-	-	-
69	SM-2	Guangdong	Chicken	Salmonella		-	-	-	-	-
70	JL-1	Jingzhou	Chicken	Staphylococcus		-	-	-	-	-
71	JZ-1	Jingzhou	Chicken	Bacillus		-	-	-	-	-
72	JZ-2	Jingzhou	Chicken	Bacillus		-	-	-	-	-

Note: The symbol "+" denotes the presence of plaque, whereas the symbol "−" indicates the absence of plaque.

### Isolation, purification of phages and determination of their host range

A total of five bacteriophages were isolated from chicken fecal and wastewater samples obtained across five chicken farms in Jingzhou city. The lytic activities of these bacteriophages were evaluated using the double-layer agar spot assay. As presented in Table 2, among the five bacteriophages, XQ-1 showed the broadest lytic spectrum, effectively lysing 34 out of 67 APEC strains tested. Notably, phage XQ-1 demonstrated lytic activity against 8 of 13 APEC serotype O1 strains, 4 of 9 APEC serotype O2 strains, 6 of 11 serotype O78 strains, and 10 belonging to other serotypes. In consideration of this, bacteriophage XQ-1 was selected for subsequent experimental investigation. Since bacteriophage XQ-1 specifically targeted APEC JZ-1 strain, we further characterized the antimicrobial resistance profile of this host strain. As summarized in Table 3, APEC JZ-1 exhibited a multidrug-resistant profile, demonstrating resistance to the majority of β-lactams antibiotics (except ceftriaxone and ampicillin), doxycycline, and erythromycin.

**Table 3:** Antimicrobial resistance phenotype of APEC JZ-1.

Drug Class	Antimicrobial Agent	Zone Diameter (mm)	Interpretation
β-lactams	Cefoperazone	16.1	I
	Ceftriaxone	32.2	S
	Ceftazidime	7.1	R
	Cephalexin	13.6	R
	Cefradine	16.5	I
	Cefazolin	13.2	R
	Penicillin	0.0	R
	Ampicillin	27.6	S
	Carbenicillin	0.0	R
	Oxacillin	0.0	R
Aminoglycosides	Gentamicin	31.5	S
	Kanamycin	23.2	S
	Neomycin	26.4	S
Tetracyclines	Doxycycline	0.0	R
	Tetracycline	15.5	I
Macrolides	Erythromycin	0.0	R

### Morphology and genomic characteristics of bacteriophage XQ-1

**Fig. 2:**
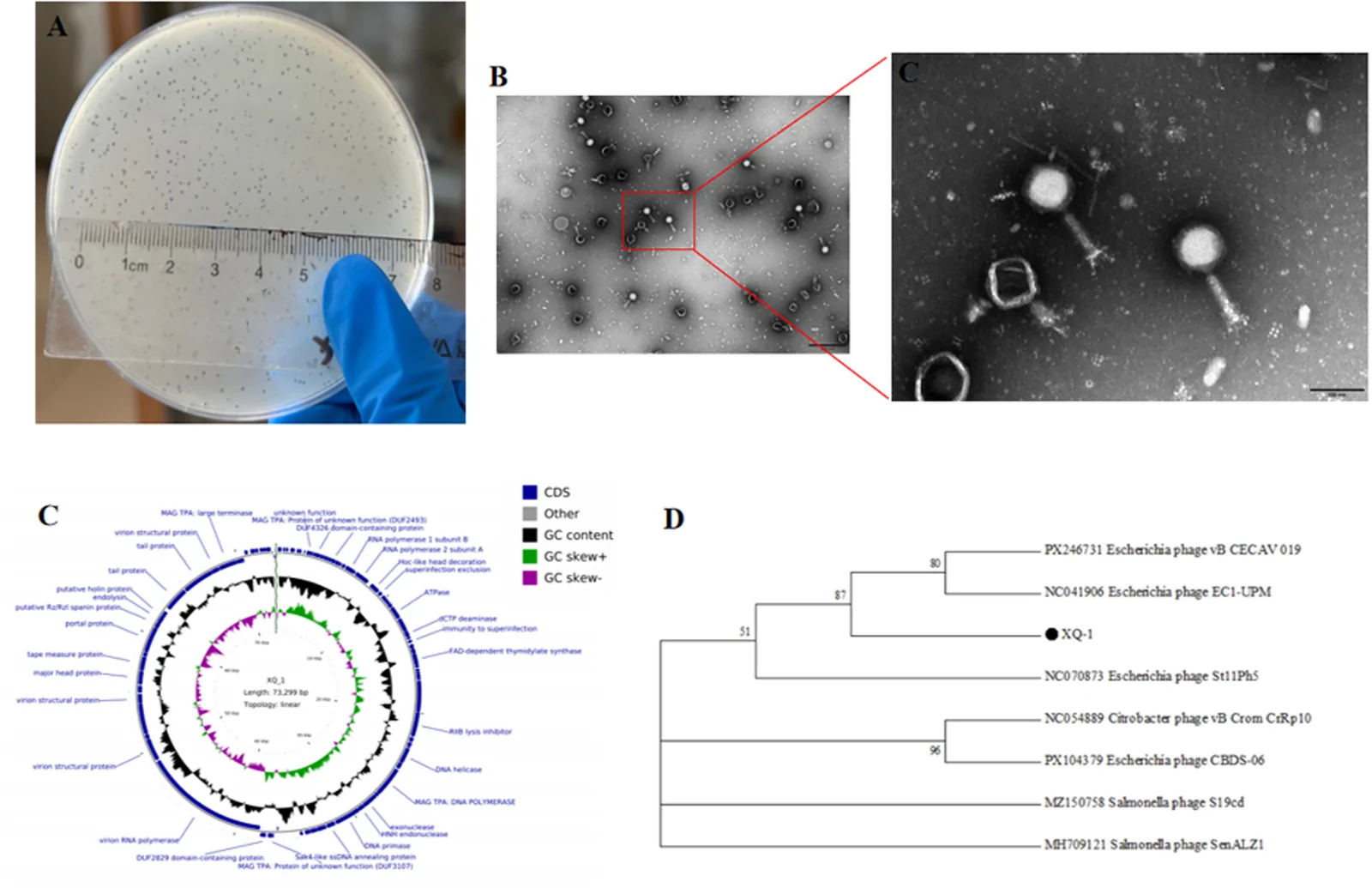
Morphology and genomic characteristics of bacteriophage XQ-1. (A) The plaque of bacteriophage XQ-1 on LB agar plate, clear zones observed on bacterial lawns signify lysis caused by phage activity. (B) TEM morphological feature of bacteriophage XQ-1, the scale bar = 100 nm. (C) The whole genome map of bacteriophage XQ-1. Circles represented (from the inside to outside) scale, GC skew, GC content, respectively. And the final rings represented the positions of CDS, tRNA, and rRNA within the genome. (D) Phylogenetic analysis of bacteriophage XQ-1. Neighbor-joining phylogenetic tree was constructed based on the complete genome of bacteriophage XQ-1 and other reference strain (Kimura 2-parameter model, bootstrap replicates = 1000).

As illustrated in Fig. 2**A**, bacteriophage XQ-1 produced distinct and circular plaques with an average diameter of 1 mm following 16 h of incubation at 37 °C on double agar plates. Further transmission electron microscopy (TEM) showed that the capsid bacteriophage XQ-1 exhibited a morphology akin to a regular hexagon, possessing a diameter ranging from 80 to 100 nm. Its tail measured between 110 and 130 nm in length, characteristics that classify it as a representative member of the Siphoviridae family (Fig. 2**B**).

Sequencing analysis revealed that the genome of bacteriophage XQ-1 consisted of double-stranded DNA, with a total length of 73, 299 bp and a G+C content of 42.88%. The genome encodes 90 open reading frames, and no genes associated with virulence or antibiotic resistance were identified (Fig. 2**C**). The genomic sequence and its basic information of bacteriophage XQ-1 has been deposited in the GenBank database (accession number PZ347661). Comparative analysis using the BLAST database indicated that the XQ-1 genome shared the highest nucleotide sequence similarity (94.48%) with the *E. coli* phage vB_CECAV_019 (GenBank accession number PX246731). Furthermore, phylogenetic analysis positioned XQ-1 in close proximity to bacteriophages belonging to the *Gamaleyavirus* genus, where it constituted a distinct evolutionary lineage (Fig. 2**D**).

### Optimal multiplicity of infection for bacteriophage XQ-1

**Fig. 3:**
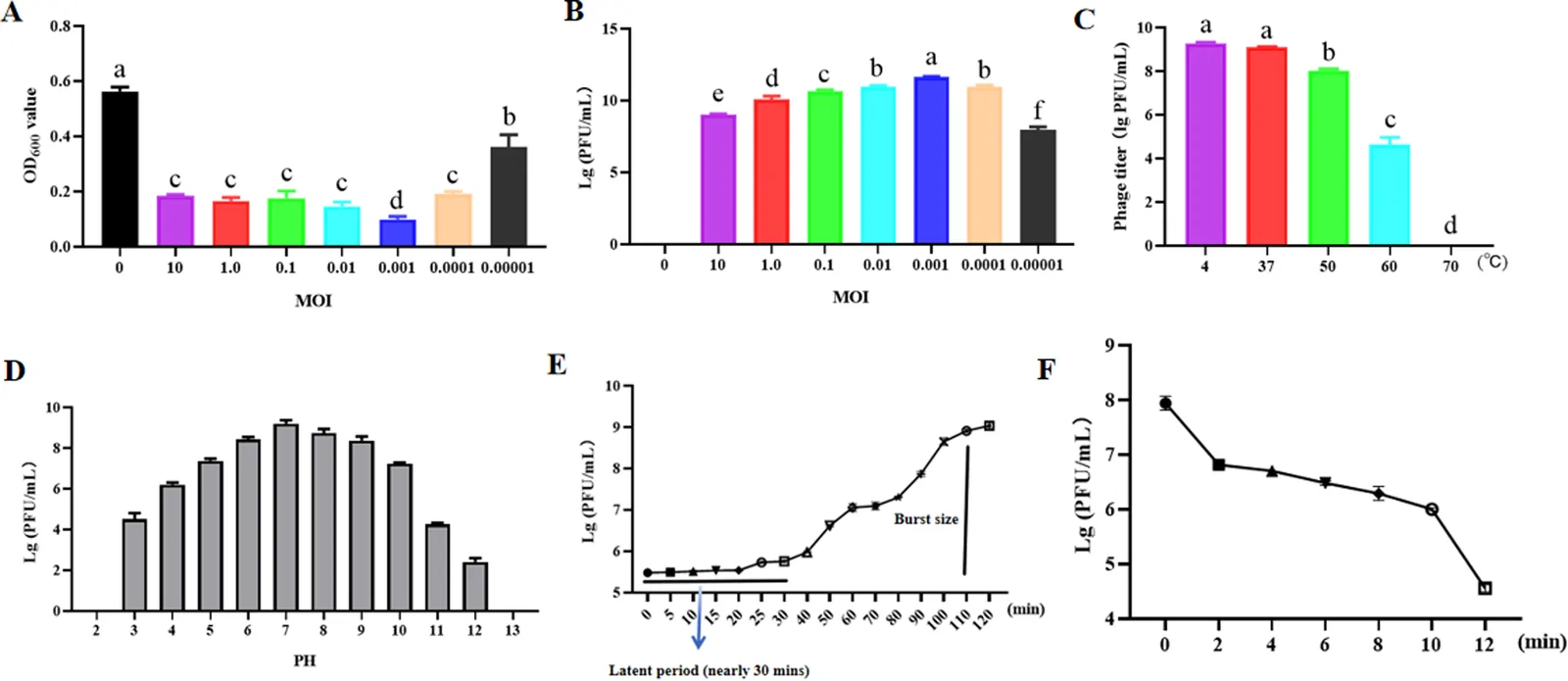
Biological characteristics of bacteriophage XQ-1. (**A-B**) Optimal MOI for bacteriophage XQ-1. Both the lowest OD_600_ measurement of host bacteria and maximal phage titer were obtained at an MOI of 0.001. (**C**) The temperature stability of bacteriophage XQ-1. Following one hour of exposure to varying temperatures, the phage demonstrated stability at 37 °C and 50 °C; however, a significant decrease in viral titer was observed at 60 °C and 70 °C. (**D**) The pH stability of bacteriophage XQ-1. Following one hour of exposure to varying pH, the phage demonstrated comparable stability across a pH range of 4 to 10. (**E**) One step growth curve of bacteriophage XQ-1. The latent period was nearly 30 min, and the burst size was determined to be 348 PFU/cell. (F) Adsorption activity of bacteriophage XQ-1 to the host cells. After adsorption at different time points, the supernatant was collected following centrifugation for the purpose of titer assessment. Error bars depict the standard error of the mean (SEM), and values marked with different superscripts denote statistically significant differences (*P* < 0.05).

To determine the optimal multiplicity of infection (MOI), the host bacterial strain APEC JZ-1 was adjusted to a final concentration of 1.0 × 10^10^ CFU/mL. Purified bacteriophage XQ-1 and host bacteria were mixed at various MOIs (10.0, 1.0, 0.1, 0.01, 0.001, 0.0001, and 0.00001) in separate tubes, respectively. An appropriate volume of LB broth was added to each group to ensure consistent culture medium volumes across all samples. For each MOI, three independent biological replicates were prepared in separate tubes. A negative control (LB broth without bacteria or phage) and a positive control (bacteria without phage, treated with equal volume of PBS) were included in each independent experiment to establish baseline OD_600_ value. Each test tube was incubated at 37 °C with shaking at 200 rpm for 2 h. Subsequently, the proliferation of APEC in each group was assessed by measuring the OD_600_ value. Following incubation, the mixtures were centrifuged, and the supernatants were collected and filtered through a 0.22 μm membrane filter. The bacteriophage titers in each group were then determined using a double layer agar plate assay. As shown in Fig. 3**A**, bacterial growth (OD_600_ value) was significantly inhibited across all MOI groups compared to the positive control (MOI=0) (*P* < 0.05). Moreover, the OD_600_ value of group (MOI=0.001, 0.06±0.02) was lowest among other experimental groups. Correspondingly, the phage titer was highest at MOI = 0.001, reaching 4.73±0.31 × 10^11^ PFU/mL (Fig. 3**B**), which was significantly higher than other groups (*P* < 0.05). Therefore, MOI = 0.001 was selected as the optimal MOI for subsequent experiments.

### The temperature and pH stability of bacteriophage XQ-1

As depicted in Fig. 3**C**, exposure to temperatures of 4 °C and 37 °C did not lead to a significant reduction in bacteriophage titer, with titers measured at 10^9.32±0.22^ PFU/mL and 10^9.1±0.15^ PFU/mL, respectively. After a one-hour incubation at 50 °C, the bacteriophage titer decreased by approximately one logarithmic scale. At 60 °C, the titer declined by more than four orders of magnitude, and complete inactivation of the bacteriophages was observed at 70 °C. As shown in Fig. 3**D,** the APEC bacteriophage XQ-1 demonstrated considerable stability across a pH range of 4–10, maintaining a titer exceeding 10^6^ PFU/mL. Conversely, the phage’s potency markedly declined as pH values above 11 or at pH values equal to or below 3. Taken together, these findings demonstrated that bacteriophage XQ-1 exhibited a strong resilience to variations in temperature and pH levels.

### One step growth and adsorption activity

According to the one step growth curve analysis, the bacteriophage XQ-1 showed a latent period of approximately 30 min and attained the plateau phase around 110 min post-infection, with a burst size estimated at 348 plaque-forming units per cell (Fig. 3**E**). Moreover, the adsorption rate of this bacteriophage was 92.77% within 2 min, 93.97% within 4 min, 96.60% within 6 min, 97.71% within 8 min, 98.88% within 10 min, and 99.95% within 12 min (Fig. 3**F**). In summary, bacteriophage XQ-1 exhibited lytic activity characterized by rapid adsorption, an intermediate latent period, and a substantial burst size. These attributes render it a potential candidate for utilization in fields such as bacteriophage therapy, food safety control, and environmental decontamination.

### In vitro disinfection ability of bacteriophage XQ-1 targeting APEC JZ-1 strain

**Fig. 4:**
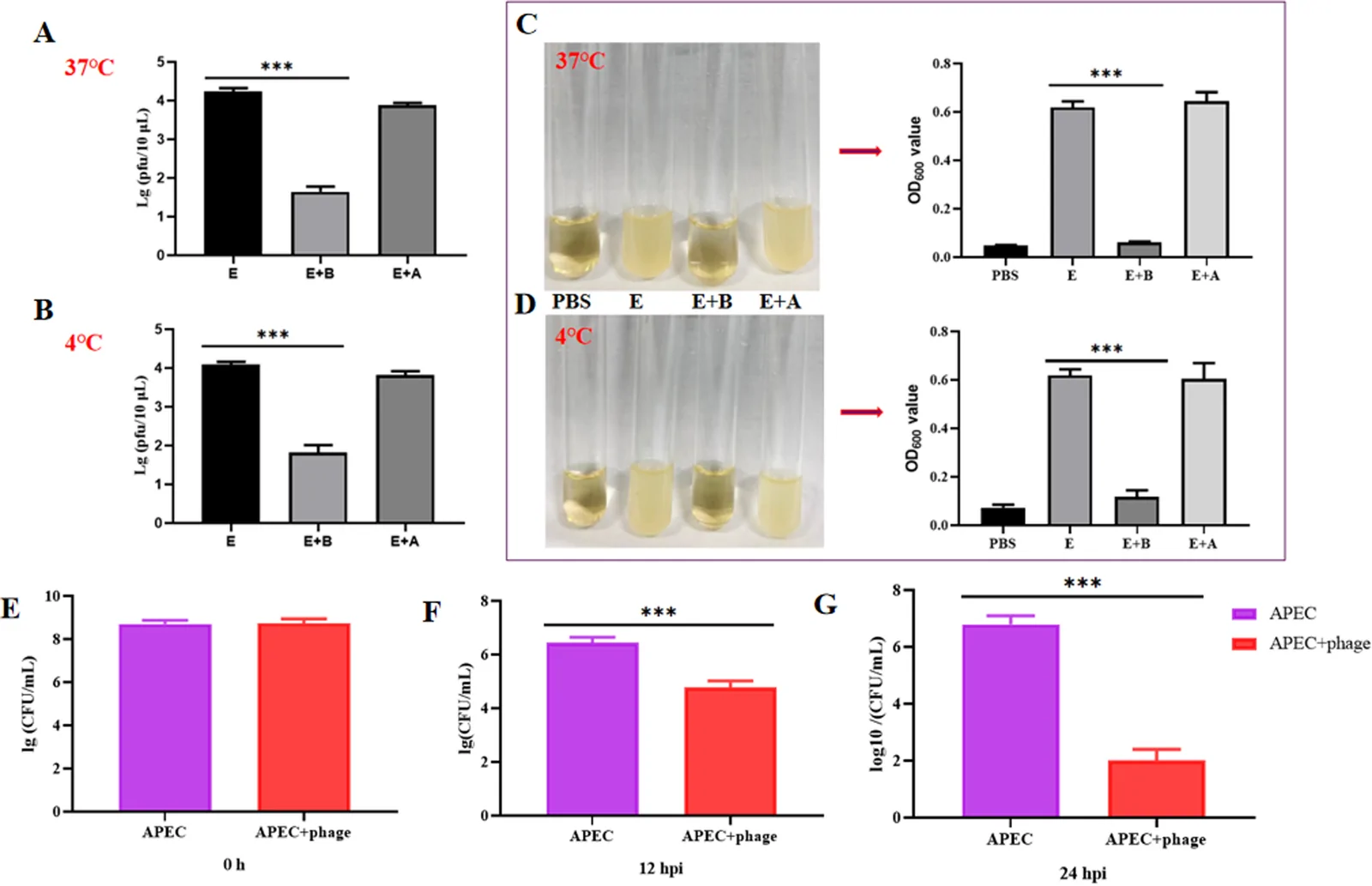
*In vitro* disinfection ability of bacteriophage XQ-1 targeting APEC JZ-1 strain. (A-D) Reduction of APEC JZ-1 levels in artificially contaminated solid surfaces at different temperatures by bacteriophage XQ-1. (A-B) The bacterial loads of APEC JZ-1 across various groups under different temperature conditions were quantified and expressed as plaque-forming units per milliliter (PFU/mL). (C-D) The visual characteristics and OD_600_ measurements of bacterial suspension turbidity across various groups under different temperature conditions. E, E+P, and E+A represented the APEC, APEC treated with bacteriophage JZ-1, and APEC treated with antibiotics, respectively. (E-G) Reduction of the bacterial loads of APEC JZ-1 on the eggshells at different time points. Error bars depict the standard error of the mean (SEM), and values marked with different superscripts denote statistically significant differences (*P* < 0.05).

In the present study, the surface of a petri dish was utilized as a substrate to model bacterial contamination on solid surfaces. As illustrated in Fig. 4, incubation at either 37 °C or 4 °C followed by treatment with bacteriophage XQ-1 resulted in a significant reduction in colony counts of the plates (Fig. 4**A and B**). Consistently, after bacterial growth in each experimental group, the OD_600_ value of the bacteria treated with bacteriophage XQ-1 was markedly lower than that of the control group (Fig. 4**C and D**).

Next, the efficacy of bacteriophage XQ-1 against APEC JZ-1 on artificially contaminated eggshells was assessed. At 0 hour post-treatment, no significant difference in bacterial load was observed between the phage-treated and control groups (Fig. 4**E**). However, by 12 h, the phage-treated group showed a approximately 20-fold reduction (Fig. 4**F**). By 24 hours, the bacterial load had further declined, exhibiting a reduction ranging from 1000 to 10,000-fold relative to the control (Fig. 4**G**). Collectively, these findings demonstrated that bacteriophage XQ-1 exhibited potential antibacterial efficacy against APEC JZ-1 on both solid media and eggshells.

### In vivo therapeutic efficiency of bacteriophage XQ-1 targeting APEC JZ-1 strain

First, we established that the median lethal dose (LD_50_) of APEC JZ-1 for one-week-old broiler chickens was 2 × 10^8^ CFU/chicken. The clinical manifestations observed in broiler chickens challenged with APEC predominantly included mental lethargy, retraction of the head, eyelid closure, ruffled feathers, drooping wings, and a tendency to cluster together as a response to cold. Moreover, lesions observed in the organs and tissues of broiler chickens encompass pericarditis, pronounced hepatic pathology, and hemorrhagic inflammation of the intestines.

**Fig. 5:**
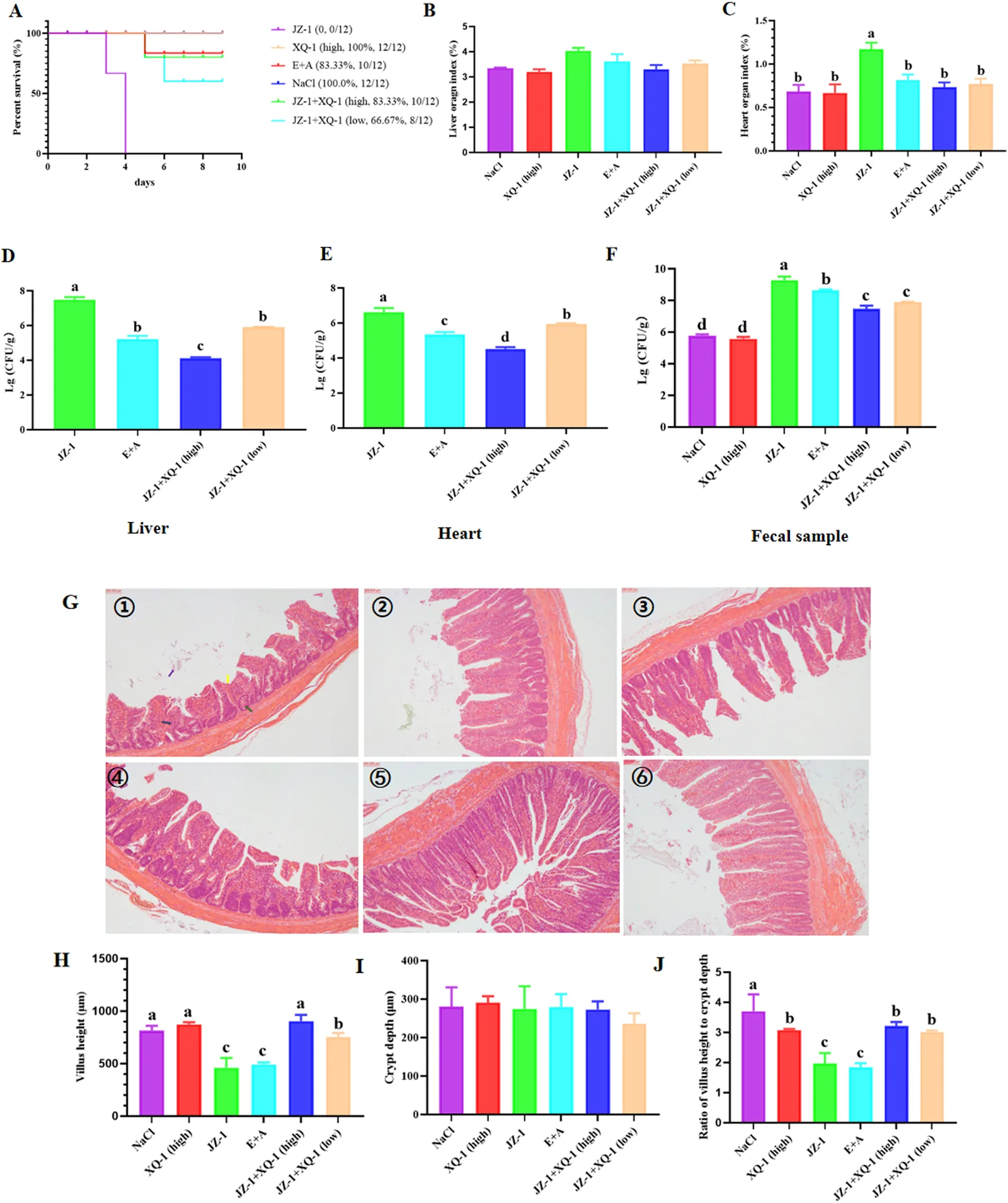
*In vivo* therapeutic efficiency of bacteriophage XQ-1 targeting APEC JZ-1 strain. (**A**) The survival rate of broiler chickens in various groups. (**B-C**) The organ indexes of live and heart in broiler chickens across various groups. (**D-**F) The bacterial load of APEC XQ-1 in liver, heart, and fecal samples of broiler chickens across different groups. (**G**) Histopathological examination of ileum tissue samples of broiler chickens across experimental groups (HE, × 100). (**H-J**) The villus height, crypt depth, and the ratio of villus height to crypt depth of ileum tissue samples across different groups. Error bars depict the standard error of the mean (SEM), and values marked with different superscripts denote statistically significant differences (*P* < 0.05).

Next, the therapeutic effectiveness of bacteriophage XQ-1 in treating APEC infection was assessed using chicken models. At the conclusion of the *in vivo* experiment, challenge with APEC JZ-1 resulted in a 100% (12/12) mortality rate in the broiler chickens, however, administration of either a low or high dose of bacteriophage XQ-1 significantly enhanced the survival rates of the APEC-challenged subjects (Fig. 5**A**). Notably, significant tissue damage was observed in the liver, heart, and intestines; in contrast, treatment with bacteriophage XQ-1 alleviated or completely resolved these pathological alterations in broiler chickens infected with APEC (Supplementary Fig. 1A-C).

Although no significant differences were observed in liver organ index among the various groups (*P* > 0.05) (Fig. 5**B**), the heart organ index in the “APEC JZ-1” group was significantly elevated compared to the other groups (*P* < 0.05) (Fig. 5**C**). Moreover, bacterial loads in the heart and liver tissue, as well as fecal sample of the “APEC JZ-1” group were significantly higher than those in the other groups. Notably, the bacterial loads in the “JZ-1+XQ-1 (high)” group were significantly lower than those observed in the antibiotic-treated group (Fig. 5**D-F**).

In addition, we examined the pathological changes in the ileum tissue samples of broiler chickens (Fig. 5**G**). The results showed that the epithelial cells and intestinal microvilli were well-preserved and orderly organized in the control group. In comparison to the control group, infection with APEC JZ-1 led to significant damage to the mucosal tissue, showing partial destruction of the villi and loss of the surface epithelial layer. This damage exposed the lamina propria and was accompanied by infiltration of inflammatory cells. Moreover, the crypts (indicated by the green arrow) appeared shortened and more widely spaced. A small amount quantity of shed epithelial debris (marked by the purple arrow) was also observed within the intestinal lumen (Fig. 5**G-①**). However, treatment with bacteriophage XQ-1 or antibiotics effectively reduced the tissue damage in the ileum samples (Fig. 5**G**). Additional quantitative measurement showed that APEC infection markedly decreased the villus height and the villus height to crypt depth ratio in the chicken ileum compared to the control group (Fig. 5**H-J**). Compared with the APEC-infected broiler chickens, treatment with bacteriophage XQ-1 but not antibiotic significantly alleviated the adverse effects induced by APEC infection, as reflected by restored villus height and villus height-to-crypt depth ratio in the ileum (Fig. 5**H-J**).

## Discussion

APEC causes avian colibacillosis, a disease that causes considerable economic losses to the poultry sector (Xu et al., 2026). The serotypes of APEC are very complex and varied, and the lack of cross-protection among serotypes creates major difficulties for effective prevention and control strategies (Kathayat et al., 2021). The excessive use of antibiotics has led to the widespread development of multidrug-resistant APEC, the presence of drug residues, and imbalances in the microbial communities within livestock environments, all of which pose significant risks to human health (Hou et al., 2024; Zakhour et al., 2024). As regulations increasingly restrict or prohibit the use of antibiotics, there is an urgent need for effective alternatives. Bacteriophages, which are highly specific, safe, and have a low likelihood of causing resistance, present a promising option (D'Amico-Willman et al., 2026).

In the present study, a novel bacteriophage designated XQ-1 was isolated from a wastewater sample collected at a chicken farm, this bacteriophage demonstrated the capacity to lyse a drug-resistant APEC strain (JZ-1). Additionally, bacteriophage XQ-1 exhibited potent bactericidal effects against APEC JZ-1 both *in vivo* and *in vitro.* Importantly, bacteriophage XQ-1 displayed a broad host range, effectively lysing more than 50% (34/67) of the tested APEC strains, encompassing multiple serotypes. The host range of XQ-1 was notably broader than that reported for several previously characterized bacteriophages, including bacteriophage vB_Ecos-TPF103dw, which lysed only O103 and some O26 *E. coli* strains (Campos et al., 2025); PEC9, that targeted O1 (9/20) and O2 (6/20) APEC strains (Yao et al., 2023); and bacteriophage UPEC04, which inhibited 17.9% (10/56) of APEC strains (Kazibwe et al., 2020). However, the host range of XQ-1 was somewhat narrower than that of bacteriophage vB_EcoM_P3322, that exhibited lytic activity against 65 out of 97 strains spanning five APEC serotypes (Huang et al., 2025). Nevertheless, these findings suggest that XQ-1 represents a promising candidate for the development of bacteriophage-based biocontrol strategies targeting APEC within poultry production systems.

Further morphological and genomic characterization confirmed that XQ-1 was a long-tailed bacteriophage belonging to the *Siphoviridae* family. Most importantly, the complete absence of virulence and antibiotic resistance genes within the XQ-1 genome represents a significant safety attribute for prospective therapeutic application. This characteristic is consistent with contemporary regulatory requirements for bacteriophage therapy in both human and veterinary medicine (Kortright et al., 2019). In addition to its genomic safety, bacteriophage XQ-1 exhibited advantageous biological characteristics conducive to practical application. It demonstrated a short adsorption time, achieving over 92% adsorption within two minutes, and a relatively brief latent period of approximately 30 minutes. Previous studies have reported the latent periods of APEC-specific bacteriophages ranging from 10 to 40 minutes (Yao et al., 2023; Huang et al., 2025; Li et al., 2025). However, bacteriophage XQ-1 exhibited superior adsorption efficiency when compared to other documented APEC-specific bacteriophages, including bacteriophage PEC9, which had an adsorption rate of 33.8% within 2 min (Yao et al., 2023), and bacteriophage vB_EcoM_GXW16 that exhibited a 25.52% adsorption rate within the same timeframe (Xu et al., 2026). The rapid adsorption coupled with effective lytic characteristic of bacteriophage XQ-1 presents significant advantages for therapeutic applications, facilitating prompt reduction of bacterial populations prior to full activation of the host immune response or the onset of extensive tissue damage.

Notably, bacteriophage XQ-1 maintained stable lytic activity over an extensive temperature range (4–50 °C) and a broad pH spectrum (3-11). Such stability is particularly advantageous for poultry production settings, where ambient temperatures may vary substantially and surfaces are frequently subjected to diverse cleaning agents with different pH levels. In comparison to other previously characterized APEC phages, which typically lose activity at pH values below 4 or above 10 (Huang et al., 2025; Khan et al., 2025; Li et al., 2025), bacteriophage XQ-1 demonstrated markedly enhanced pH tolerance. This pronounced environmental resilience in lytic activity suggests that XQ-1 holds potential not only as an injectable therapeutic agent but also as an aerosolized or spray-applied disinfectant for use in poultry housing, egg sanitization, and equipment decontamination.

The *in vitro* disinfectant tests offered direct evidence of this potential. When applied to artificially contaminated plastic surfaces, a 10-min treatment with bacteriophage XQ-1 at either 37 °C or 4 °C significantly lower bacterial counts compared to untreated samples. More importantly, bacteriophage XQ-1 effectively decontaminated APEC JZ-1 on eggshell surfaces, reducing the bacterial load by 1000 to 10,000 times within 24 h. Contamination of eggshells is a critical entry point for APEC in many poultry operations, as hatchery infections often originate from fecal contamination on egg surfaces (Adams et al., 2025). The ability to apply phages as a spray or dip during egg processing could provide a non-chemical, residue-free method of intervention, which is especially beneficial for organic or antibiotic-free production systems.

The *in vivo* therapeutic efficacy of bacteriophage XQ-1 was particularly striking. Administration of an APEC challenge at a concentration of 10^9^ CFU per animal resulted in complete mortality within the observation period, accompanied by severe pathological manifestations including pericarditis, hepatitis, and hemorrhagic intestinal lesions. Treatment with both high-dose and low-dose treatment of bacteriophage XQ-1 significantly enhanced survival rates, decreased bacterial loads in the heart, liver, and fecal samples, and alleviated tissue damage. Significantly, bacteriophage XQ-1 treatment demonstrated superior efficacy compared to traditional florfenicol antibiotic therapy across several parameters, including intestinal villus height and the villus height-to-crypt depth ratio. Given that intestinal integrity is a critical factor influencing nutrient absorption and disease resistance in poultry (Adedokun and Olojede, 2019; Azizi et al., 2026), the enhanced preservation of intestinal morphology observed with bacteriophage XQ-1 may be attributed to its targeted action against APEC, which preserves the broader intestinal microbiota. In contrast, antibiotic treatments frequently induce collateral dysbiosis (Wintachai et al., 2024).

Despite these promising findings, several limitations of the present study should be acknowledged. Firstly, the *in vivo* experiments were conducted over a relatively brief observation period of one week, and this study did not include direct analyses of gut mictobiota composition. Extended longitudinal studies incorporating 16S rRNA sequencing or metagenomic analysis of fecal samples are warranted to evaluate long-term outcomes such as phage resistance development and gut microbial restoration. Secondly, the use of intraperitoneal injection as the challenge route may not accurately mimic natural infection pathways; thus, future investigations should consider assessing the protective efficacy of oral phage administration. Thirdly, reliance on monophage therapy may elevate the risk of resistance emergence, suggesting that the application of phage cocktails or synergistic combinations with subtherapeutic antibiotic doses merits exploration. Fourthly, the in vitro disinfection assays were conducted under controlled laboratory conditions devoid of organic matter, such as litter, feces, or feed residues, which are prevalent in farm environments. Future experimental designs should integrate these factors to more accurately simulate field conditions. Finally, the host range of bacteriophage XQ-1 was restricted, as it lysed only about 50% (34 out of 67) of the tested APEC isolates. This finding suggests that monophage therapy may not offer adequate coverage against the diverse strains encountered in the field. Consequently, it is necessary to isolate additional lytic bacteriophages with complementary host specificities and combine them with XQ-1 to develop effective phage cocktails for broader therapeutic application.

## Conclusion

In summary, a novel virulent bacteriophage designated XQ-1 was successfully isolated and thoroughly characterized. Bacteriophage XQ-1 exhibits advantageous biological attributes, including a broad host spectrum encompassing multiple APEC serotypes, rapid adsorption kinetics, a brief latent period, a high burst size, and notable stability across a wide temperature range and pH spectrum. Genomic analysis revealed the absence of genes associated with virulence or antibiotic resistance, thereby affirming its safety profile. Crucially, bacteriophage XQ-1 demonstrated strong antibacterial activity both in vitro, serving effectively as a disinfectant on surfaces and eggshells, and in vivo, where it significantly enhanced survival rates, decreased bacterial burdens in organs, and alleviated intestinal pathology in APEC-infected chicken models. Collectively, these results suggest that bacteriophage XQ-1 represents a promising and safe alternative to conventional antibiotics for the prevention and treatment of APEC infections in poultry.

## Funding

This research was supported by the Yunnan Provincial Department of Science and Technology Major Science and Technology Special Program (Grant No. 202602AE090031), the Yunnan Provincial Department of Science and Technology Rural Revitalization Science and Technology Special Project (Grant No. 202604BI090001), the Open Project of Yunnan Provincial Key Laboratory of Tropical and Subtropical Animal Viral Diseases (Grant No. 2025RW001), the Hubei Provincial Science and Technology Plan Project (Grant No. 2024BBB066), the the modern agricultural industry technology system of Hubei Province, poultry and egg industry technology system (Grant No. 2025HBSTX4-04), and the College Student Innovation and Entrepreneurship Training Program Project in Yangtze University (Grant No. Yz2025087 and Yz2025084).

## Institutional review board statement

All animal experiments in the present study were conducted in accordance with the approval granted by the Animal Ethics Committee of the Yangtze University (Approval Number: 202601011).

## Author contributions

**Yuqing Duan** was responsible for conducting the original research, including bacterial and phage isolation, investigating the biological characteristics of phages. She was also involved in the data analysis and the preparation of the original draft. **Changchun Gao** was involved in sample collection , performing the statistical analysis, and he participated in chicken animal experiment, Writing-original draft, and Writing-review and editing. **Xi Yang** participated in fecal sample collection , bacterial isolation and molecular identification , and formal analysis of these strains. moreover, she was also involved in the preparation of original draft. **Pei Zhu** was involved in the related experiments and formal analysis of bacteriophage electron microscopy; meanwhile, she participated in the preparation of the map of phage genome, and the preparation of original draft. **Jinping Wang** was involved in the experiment and formal analysis of bacteriophage electron microscopy, genome assembly, and the preparation of original draft and revised version. **Yun Zhou** was involved in clinical sample collection, bacterial isolation, and formal analysis, as well as the preparation of the original draft. **Zihan Yang** was involved in clinical sample collection, the isolation of bacteria and bacteriophage, as well as the exploration of the biological characteristics of phage and formal analysis. **Yitong Guo** was involved in clinical sample collection, the isolation of bacteria and bacteriophage, as well as the exploration of the biological characteristics of phage and formal analysis. **Guangming Tian** participated in the collection of clinical samples, the formal analysis of genomic characteristics of phage genome, and the animal experiments, as well as the funding (Grant No. 2024BBB066 and 2025HBSTX4-04). **Jiay ing Liu** was involved in clinical sample collection and phage isolation, formal experimental and analysis of drug susceptibility test of bacteria, statistical analysis, and the preparation of original draft. **Xiaowei Fang** pariticipated in . clinical fecal sample collection, isolation and molecular identification of APEC, animal experiment of APEC, and the preparation of original draft. **Mengting Zuo** was involved in the formal analysis of the genomic characterisitic of phage genome, designation of the animal experiment, the preparation of original draft and the revised version. Lei Tan designated the whole experimental processes, formal analysis of the biological features of phage, preparation of the original draft and the revised version, and received the funding (Grant No. 2025RW001, Yz2025087 and Yz2025084) Jun Yao i ndependently verified the reproducibility of all data and validated the accuracy of the data analysis. He also provided the overall guidance for the research projects, and provided the funds (202602AE090031 and 202604BI090001) to support this research. Moreover, He was responsible for the preparation of the original draft and the revised version.

## Disclosures

Changchun Gao is affiliated with Beijing Huadu Yukou Poultry Industry Co., Ltd. The other authors state that they have no conflicts of interest regarding this paper.

## Footnotes

Supplementary material associated with this article can be found, in the online version, at doi:10.1016/j.psj.2026.107600.
